# Age of Information Analysis for Multi-Priority Queue and Non-Orthoganal Multiple Access (NOMA)-Enabled Cellular Vehicle-to-Everything in Internet of Vehicles

**DOI:** 10.3390/s24247966

**Published:** 2024-12-13

**Authors:** Zheng Zhang, Qiong Wu, Pingyi Fan, Qiang Fan

**Affiliations:** 1 School of Internet of Things Engineering, Jiangnan University, Wuxi 214122, China; zhengzhang@stu.jiangnan.edu.cn; 2 Department of Electronic Engineering, Beijing National Research Center for Information Science and Technology, Tsinghua University, Beijing 100084, China; fpy@tsinghua.edu.cn; 3 Qualcomm, San Jose, CA 95110, USA; qf9898@gmail.com

**Keywords:** C-V2X, resource reservation interval, non-orthogonal multiple access, age of information

## Abstract

With the development of Internet of Vehicles (IoV) technology, the need for real-time data processing and communication in vehicles is increasing. Traditional request-based methods face challenges in terms of latency and bandwidth limitations. Mode 4 in cellular vehicle-to-everything (C-V2X), also known as autonomous resource selection, aims to address latency and overhead issues by dynamically selecting communication resources based on real-time conditions. However, semi-persistent scheduling (SPS), which relies on distributed sensing, may lead to a high number of collisions due to the lack of centralized coordination in resource allocation. On the other hand, non-orthogonal multiple access (NOMA) can alleviate the problem of reduced packet reception probability due to collisions. Age of Information (AoI) includes the time a message spends in both local waiting and transmission processes and thus is a comprehensive metric for reliability and latency performance. To address these issues, in C-V2X, the waiting process can be extended to the queuing process, influenced by packet generation rate and resource reservation interval (RRI), while the transmission process is mainly affected by transmission delay and success rate. In fact, a smaller selection window (SW) limits the number of available resources for vehicles, resulting in higher collisions when the number of vehicles is increasing rapidly. SW is generally equal to RRI, which not only affects the AoI part in the queuing process but also the AoI part in the transmission process. Therefore, this paper proposes an AoI estimation method based on multi-priority data type queues and considers the influence of NOMA on the AoI generated in both processes in C-V2X system under different RRI conditions. Our experiments show that using multiple priority queues can reduce the AoI of urgent messages in the queue, thereby providing better service about the urgent message in the whole vehicular network. Additionally, applying NOMA can further reduce the AoI of the messages received by the vehicle.

## 1. Introduction

With advanced IoV technology, the development of intelligent transportation systems has facilitated various applications such as automated navigation, collision warning, and multimedia entertainment [1,2,3]. Due to the high-speed mobility of vehicles, ensuring timely data access through user requests is crucial [4,5,6]. In the traditional request-based approach, users connect their base stations, via which they can access the data center to retrieve the requested data [7,8,9,10,11,12]. However, this method suffers from end-to-end delays and limited backhaul bandwidth [13,14,15,16]. To address these issues, C-V2X proposes an interface called PC5 for communication between autonomous vehicles. PC5 offers two resource allocation methods: Mode 3, where user equipment (UE) requests time and frequency-domain transmission resources from the eNodeB, and Mode 4, where UE autonomously selects resources without involving the cellular infrastructure. Mode 4 not only eliminates the limited coverage drawback but also minimizes interaction between base stations and vehicles, thereby resolving excessive delays and overhead [17].

In Mode 4, vehicles autonomously select communication resources using the SPS protocol based on SPS, allowing vehicles to choose among RRI. However, this increases the collision probability when multiple vehicles occupy the same resources with the same message transmission interval, leading to higher block error rate (BLER) [18]. NOMA is a potential solution for C-V2X communication, promising to enhance spectrum efficiency and handle large-scale vehicle communications, mitigating latency and packet reception probability degradation caused by high vehicle density [19,20]. Successive interference cancellation (SIC) is a well-known multi-user detection technique used to extract overlapping signals, decoding different power levels of received signals occupying the same resource. Thus, the high power signals no longer interfere with other low-power signals after decoding, improving the signal-to-interference-plus-noise ratio (SINR) of low-power signals block error rate (BLER) [21,22,23,24].

Some studies have examined the effectiveness of NOMA applied in C-V2X. In [25], a NOMA receiver based on SIC and joint decoding was proposed to reduce BLER compared to traditional orthogonal multiple access (OMA) methods. In [26], TAKESHI et al. introduced SPS-NOMA based on uplink non-orthogonal multiple access to improve the SIC under broadcast scenarios and alleviate channel congestion. In [27], Utpal et al. proposed a model with a large-scale MIMO Jacobi detection algorithm for the PHY layer of C-V2X, enhancing reliability by reducing bit error rate compared to existing PHY layer frameworks. These works demonstrated improvement of NOMA in terms of reliability and transmission delay in the C-V2X system.

It is worth noting that these two metrics are often in trade-off, where an increase in reliability performance may come at the cost of increased delay. Therefore, a new metric is necessary to comprehensively reflect both reliability and latency performance, such as AoI. A lower average AoI indicates lower latency with the same reliability and higher reliability with the same delay Therefore, a new metric like AoI is necessary as it provides a comprehensive reflection of both reliability and latency performance. A lower average AoI indicates either lower latency with the same reliability or higher reliability with the same delay, thereby better measuring the timeliness of information and the overall performance of the system [28,29,30]. In [31], Peng et al. adopted AoI to evaluate the MAC layer performance of the C-V2X sidelink, proposing a Piggyback-based cooperative method for vehicles to inform each other of potential resource occupation, reducing collisions and exhibiting good AoI performance. In [32], Zoubeir et al. presented a resource allocation problem based on NOMA, optimizing resource allocation to provide minimum AoI and high reliability for vehicle safety information.

However, the aforementioned studies did not consider the AoI of the packet in the queue in the C-V2X system. The packet at the receiver includes the packet generation time, which should be a key factor for the queuing process. In [33], Akar et al. investigated the freshness of information in IoT-based state update systems using the AoI performance metric. They studied discrete-time servers in multi-source IoT systems, assuming Bernoulli arrivals of information packets and universally distributed discrete phase-type service times across all sources. Their analysis of AoI under various queuing disciplines was formulated in matrix-geometric terms. In [34], Zhang et al. considered a dual-server short-block wireless communication system to ensure real-time delivery of newly generated information at a relatively high update rate to its destination. Information is generated at a relatively high update rate, encoded into two short-block queues and delivered in real-time through two parallel paths. The study based on the Markov-chain process investigated the AoI performance of the dual-queue system in the presence of block delivery errors. In [35], Liu et al. propose a hybrid TDMA and NOMA protocol that takes advantage of the two protocols and a clustering-based dynamic adjustment of the shortest path algorithm to the long-term average AoI in an unmanned-aerial-vehicle-assisted wireless-powered communication network.

The AoI of the packet generated in the queue is influenced by packet generation rates and service rates, where the service rate in C-V2X depends on the RRI [36]. However, for the same RRI, while the AoI of transmitter undergoes a short queuing time, the collision probability increases, leading to a potential large AoI at the receiver [37]. This motivates us to consider this work. In this paper, we propose an AoI calculation approach based on a multi-priority data type queue in C-V2X. We first design a queue model with four types of messages of different priorities, in which the AoI of receiver better reflects the ability to observe the status of the transmitter in a timely manner. We then consider the impact of NOMA on AoI in both processes, calculate AoI for different RRIs, and analyze the effect of multi-priority queues and NOMA on the C-V2X communication system. (The source code has been released at: https://github.com/qiongwu86/Analysis-of-the-Impact-of-Multi-Priority-Queue-and-NOMA-on-Age-of-Information-in-C-V2X, accessed on 14 July 2024). The remaining parts of this article are as follows, Section 2 provides a brief introduction to the system model. Section 3 presents a description of the proposed modeling of the approach in detail. In Section 4, we present some simulation results, followed by the conclusion in Section 5.

## 2. System Model

**Figure 1:**
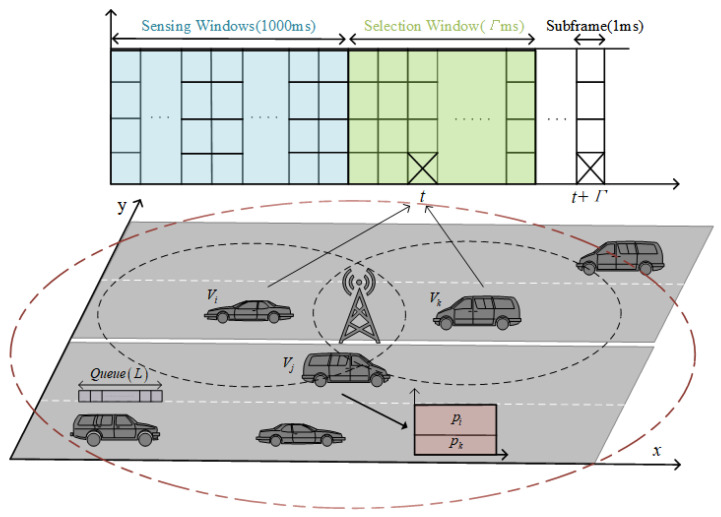
System model.

We consider the system model as shown in Figure 1. In this model, a C-V2X-based communication system is with *N* half-duplex vehicles. In C-V2X Mode 4, all communication resources are in a resource pool, with the basic unit of the resource block(RB) with a size of 180 kHz. A sub-channel typically consists of 10 RBs. The time axis is set to discrete values, with each time slot being 1ms, noted as the duration of an RB, representing the size of one sub-frame in the resource pool. In C-V2X Mode 4, there are four priority levels of message generating types. When the multi-priority queue is not empty, vehicles reserve communication resources from the resource pool. The time interval between reserved resources is defined as the RRI, commonly taking values of 20 ms, 50 ms, or 100 ms. The initial value of the reserved resources (RC), which refers to the set of resources allocated in advance to ensure efficient operation in a network, is defined as 500/RRI + rand(1000/RRI). That is to say, when the RRI is 20 ms, the output range is from 25 to 75. Moreover, due to half-duplex communication, vehicles cannot receive signals while using communication resources.

We use AoI as the performance metric for this system and calculate the AoI generated in the multi-priority queue and the transmission process. Regarding the AoI in the queue, using communication resources will change the AoI and position of messages in the queue, while not using communication resources will keep them unchanged. Regarding the AoI in the transmission process, the AoI at the receiver is updated to the AoI of the received message when communication is completed. Otherwise, it will grow over time. For simplicity, the receiver determines whether it receives the signal by comparing the SINR of the received signal with the SINR threshold as the criteria of successful transmission. When C-V2X employs OMA, it will compute SINR, where interferences mainly come from the collisions of other signals. For NOMA mode, SIC (see Equation (10) for detail) is used to improve the SINR of low-power signals in collisions, where the SINR calculated for received signals are sorted in descending order of their power and only signals with lower power than the current signal are considered as interference. Additionally, in C-V2X, for the same RRI, the AoI in the queue may be very small while the receiver’s AoI can be large. To better reflect the improvement in collisions by NOMA, it is necessary to separately calculate the AoI generated in the two processes and analyze the effect of NOMA on the C-V2X communication system.

## 3. Mathematical Model

In this section, we first establish a computational model to measure the AoI for a C-V2X system with a multi-priority message queue. Subsequently, we propose to employ NOMA based on SIC to improve the AoI of the C-V2X system. Table 1 summarizes the notations of the symbols.

### 3.1. AoI in C-V2X

(1) C-V2X Queue Model

The C-V2X Model 4 communication system includes four types of messages: high priority data (HPD), decentralized environmental notification message (DENM), signal awareness message (CAM), and miscellaneous high-density data (MHD), with the following priority order: HPD > DENM > CAM > MHD. CAM-type messages are generated periodically, while the rest of the types are triggered. The generation probability of a new CAM packet is $1/T_{c}$, where $T_{c}$ is the fixed packet generation period. The new packet generation probabilities for HPD, DENM, and MHD are expressed by a Poisson distribution:

(1) $$P\left({\mathrm{arr}}_{i,n}^{t}=k\right)=\frac{\lambda_{n}^{k}}{k!}e^{-\lambda_{n}}$$

where n∈{HPD,DENM,MHD}, and $\lambda_{n}^{k}$ represents the number of packet arrivals for each type of message in a certain time period, and *k* represents the number of data packets arriving, which is typically set to 1. For DENM and MHD, the new packets need to be retransmitted multiple times to ensure successful transmission, and this retransmission process is periodic. Thus, four corresponding first-in-first-out queues are established based on different signal types. Their queue capacity is *L*, and the queue length is *q*. When the queues meet q<L, new packets can be added and share the same transmission opportunity.

Given the transmission opportunity, vehicles can use their own reserved resources for transmission. At this point, the vehicle transmits messages from different queues based on their priority. When the *q* of the high-priority message queue is non-zero, the corresponding transmission action *s* is set to 1, while *s* of other queues with lower priorities are set to 0. Only when the length *q* of the high-priority queue is zero, can *s* of the second highest priority queue be set to 1. Here, s=1 indicates that the messages in that queue can be transmitted. Otherwise, they cannot be transmitted. Therefore, the expression for the transmission action *s* of the multi-priority queues is

(2) $$\left\{\begin{array}{cc} s_{i,H}^{t}=1,s_{i,C}^{t}=0 & q_{i,H}^{t}=1\\ s_{i,H}^{t}=0,s_{i,C}^{t}=1 & q_{i,C}^{t}\left(1-q_{i,H}^{t}\right)=1\\ s_{i,H}^{t}=s_{i,C}^{t}=0 & \;\mathrm{otherwise}\; \end{array}\right.$$

Since the generation methods of HDP, DENM, and MHD-type messages are the same, and CAM is different from them, the two priority queues with HDP and CAM-type messages can be used to represent the relationship between the representations of *s* of different queues. Let $s_{i,C}^{t}$ represent the transmission opportunity of the HPD queue at time *t* and $s_{i,H}^{t}$ represent the transmission opportunity of the CAM queue. When the *q* of all queues are zero, it indicates that no packets can be transmitted, and at this moment, all *s* values are set to 0.

(2) AoI Model

The cumulative AoI of messages in the queue is impacted by the queuing process, which is defined as the time from packet generation to transmission. It is worth noting that during the interval when the vehicle is using the reserved resource, the AoI will continuously increase. The AoI expression for each message in each queue is given by

(3) $$\varphi_{i,n}^{t+1,b}=\left\{\begin{array}{cc} \varphi_{i,n}^{t,b+1}+1 & s_{i,n}^{t}=1\\ \varphi_{i,n}^{t,b}+1 & s_{i,n}^{t}=0 \end{array}\right.$$

where *n* is the index of queues, b∈[1,q−1] represents the position of the message in the queue at time *t* for vehicle *i*, and $\varphi_{i,n}^{t,b}$ denotes the message AoI in queue *n* at position *b* for vehicle *i* at time *t*. When $s_{i,n}^{t}$ = 1, the position of all messages in the queue will be updated except for the first message. When $s_{i,n}^{t}$ = 0, all message positions in the queue remain unchanged. Here, the probability of $s_{i,n}^{t}$ = 1 represents the processing rate, depending on the 1/RRI in C-V2X. Thus, the RRI will impact the size of the AoI. In addition, when a message in the queue is transmitted, the AoI at the last position in the queue will be reset to 0.

The cumulative AoI during the communication process can be considered as the time spent to complete the transmission between the receiver and the transmitter, which is influenced by both the transmission delay and the transmission process. Additionally, since the AoI of the received message reflects the situation at the time of packet generation by the transmitter, the receiver needs to inherit it to indicate whether it communicates with the transmitter in a timely manner. The AoI expression for receiver *j* regarding transmitter *i* is given by

(4) $$\Phi_{i\to j}^{t+1}=\left\{\begin{array}{cc} \varphi_{i}^{t,1}+1 & u_{i\to j}^{t}=1\\ \Phi_{i\to j}^{t}+1 & u_{i\to j}^{t}=0 \end{array}\right.$$

where $\Phi_{i\to j}^{t}$ represents the AoI of vehicle *j* from vehicle *i* at time *t*, and $u_{i\to j}^{t}$ indicates whether the message is successfully transmitted to *j* by *i*. If $u_{i\to j}^{t}$ = 1, $\Phi_{i\to j}^{t}$ is equal to the $\varphi_{i\to j}^{t,1}$ of the highest-priority message in the queue transmitted by vehicle *i*, plus the transmission delay. If $u_{i\to j}^{t}$ = 0, $\Phi_{i\to j}^{t+1}$ is equal to $\Phi_{i\to j}^{t}$ plus the subframe size. According to Equations (3) and (4), it can be observed that transmission failure will cause a greater increase in $\Phi_{i\to j}^{t+1}$. In C-V2X model 4, each transmission failure requires waiting for an RRI interval, owing to the reserved resource. The main reason for transmission failure is a low SINR caused by collisions, which can be addressed by NOMA.

### 3.2. NOMA in C-V2X

(1) Collisions in C-V2X

In C-V2X model 4, vehicles autonomously allocate resources and transmit data in a broadcast manner. When multiple vehicles reserve resources in the neighboring time slots, they may select the same resources. Moreover, due to the broadcast nature, multiple vehicles can simultaneously communicate with the same receiver. This situation leads to collisions when multiple vehicles use the same resources to communicate with the same receiver. According to [38], the non collision probability in C-V2X model 4 can be expressed as

(5) $$\begin{array}{cc} P_{\mathrm{ncol}} & \approx {\left[1-\left[1-\prod_{i=0}^{\Gamma -1}\left(1-\frac{\pi }{1-\pi i}\right)\right]\frac{1-P_{\mathrm{rk}}}{CSR-N_{v}+1}\right]}^{N_{v}-1} \end{array}$$

where π represents the probability that a vehicle is preparing to select a resource, where it needs to satisfy the three following conditions: the vehicle queue is not empty, the RC is zero, and a new resource is being rescheduled. $P_{\mathrm{rk}}$ represents the probability of a vehicle selecting a new resource when the reselection counter resets to zero. CSR represents the total number of resources in the selection window. $N_{v}$ represents the total number of vehicles. Γ is the size of SW, which refers to the range of available time from which vehicles can select for transmission, so CSR is consistent with Γ. However, the range of Γ is small, typically being 20, 50, or 100. Thus, when a specific value of Γ is chosen, $\left[1-\prod_{i=0}^{\Gamma -1}\left(1-\frac{\pi }{1-\pi i}\right)\right]\frac{1-P_{rk}}{CSR-N_{v}+1}$ is a functions of the number of vehicles $N_{v}$. Thus, as $N_{v}$ increases, $P_{\mathrm{col}}$ also increases. Furthermore, changing the value of Γ for data transmission may reduce the collision probability, but it can also increase the waiting time of messages in the queue, leading to a larger AoI. Therefore, considering the impact of collisions, we also explore the use of NOMA to mitigate this issue while changing Γ.

(2) SINR Calculation

In C-V2X model 4, because the system uses a dynamic resource allocation approach based on the network conditions and the traffic load, the size of transmission resources is not fixed. Vehicles calculate the required bandwidth B and the transmission rate threshold $R_{th}$ for successful transmission within one subframe based on the size of the message *Q*, as follows:

(6) $$R_{\mathrm{th}}=B_{i}\log_{2}\left(1+{\mathrm{SINR}}_{\mathrm{th}}\right)$$

where $R_{\mathrm{th}}=Q$ because the time taken to complete data transmission for a message must be less than the length of one subframe, i.e., Q/R≤1. Next, perform a transformation on Equation (6); the SINR thresholdSINR_th_ is calculated based on the bandwidth and the transmission rate threshold, as shown in the following expression:

(7) $${\mathrm{SINR}}_{\mathrm{th}\;}=2^{Q_{i}/B_{i}}-1$$

Due to the time-varying distances between vehicles, the corresponding channel condition leads to varying communication rates at the receiver. Additionally, since multiple vehicles may use different communication resources within the same subframe, the receiver also receives signals at different rates within different communication resources. If the receiver receives only one signal within a resource, then the SINR between vehicle *i* and vehicle *j* at time *t* is given by

(8) $${\mathrm{SINR}}_{i\to j}^{t,n}=\frac{p_{i\to j}^{t}{\left|h_{i\to j}^{t,n}\right|}^{2}}{\sigma^{2}}$$

where $p_{i\to j}^{t}$ is the transmission power of vehicle *i*, ${\left|h_{i\to j}^{t,n}\right|}^{2}$ is the channel gain between vehicles *i* and *j* for resource *n*, and $\sigma^{2}$ is the noise power. However, due to the half-duplex resource selection scheme in C-V2X, vehicles may select the same resource block when choosing resources, leading to collisions when they use the same resource for transmission. In this case, the wireless information transmission rate is defined as

(9) $${\mathrm{SINR}}_{i\to r}^{t,n}=\frac{p_{i\to j}^{t}{\left|h_{i\to j}^{t,n}\right|}^{2}}{\sum_{m\in N_{m}}p_{m\to j}^{t}{\left|h_{m\to j}^{t,n}\right|}^{2}+\sigma^{2}}$$

where *m* represents interfering vehicles in the C-V2X scenario, and their transmission energy is denoted as $P_{m}$. It can be observed that when multiple vehicles use the same resource, the transmission rate decreases, and if the transmission rate is too low, it may result in transmission failure.

Therefore, to address this issue, we introduce NOMA based on SIC. The receiver sorts the received signals in descending order of received power, considering the maximum received power signal as the target signal and the rest as interference signals. After decoding and removing the target signal, this process is repeated for all signals to compute their SINR. Let $N_{k}=\left\{k\in N\setminus i|p_{i\to j}^{t}{\left|h_{i\to j}^{t,n}\right|}^{2}>p_{k\to j}^{t}{\left|h_{k\to j}^{t,n}\right|}^{2}\right\}$ represent the set of vehicles who has weaker received power than vehicle *i*. Then, the SINR for vehicle *i* is given by

(10) $${\mathrm{SINR}}_{i\to r}^{t,n}=\frac{p_{i\to j}^{t}{\left|h_{i\to j}^{t,n}\right|}^{2}}{\sum_{k\in N_{k}}p_{k\to j}^{t}{\left|h_{k\to j}^{t,n}\right|}^{2}+\sigma^{2}}$$

It can be seen that using NOMA in the case of a collision can reduce the interference at the target signal and increase SINR, thereby reducing the possibility of SINR to lower than the threshold. It is generally believed that ${\left|h_{i\to j}^{t,n}\right|}^{2}=c_{ij}d_{ij}$, $c_{ij}$ represents the coefficient between vehicle *i* and vehicle *j*, $d_{ij}$ denotes the distance between vehicle *i* and vehicle *j*, and η is the path loss exponent. Here, the value of $d_{ij}$ depends on the positions of vehicles *i* and *j* at time *t*.

(3) Vehicle Mobility

Consider a two-way road with a length of *D* and U/2 lanes in each direction. We establish a coordinate system where the position of vehicle *i* at time *t* is defined as $\left(x_{i}^{t},y_{i}^{u}\right)$. The origin of the coordinate system is set as the leftmost position of the road, with the *x*-axis representing the direction of vehicle movement, and the *y*-axis representing different lanes. Assuming that the vehicle updates its position periodically after a short time, the speed *V* of vehicle *i* can be considered as constant:

(11) $$x_{i}^{t+1}=x_{i}^{t}+\delta V\tau ,x_{i}^{t}\in \left[0,D\right]$$

where δ represents the direction of vehicle, and $x_{i}^{0}\in \left[0,D\right]$ is the initial position of the vehicle. Furthermore, $y_{i}^{g}$ depends on the lane index g∈[1,…,G] of vehicle *i* and is calculated as

(12) $$y_{i}^{g}=gd_{y}-y_{0}$$

where $d_{y}$ is the width of one lane, and $y_{0}$ represents the distance from vehicle *i* to the edge of the lane. Typically, $y_{0}$ is taken as $1/2d_{y}$, which is half of the lane width.

## 4. Numerical Results and Discussion

We have conducted the simulations to demonstrate our work based on the simulation tool, i.e., MATLAB R2021b [39]. In this section, we present the simulation results by comparing AoI in C-V2X under NOMA and OMA, validating the impact of NOMA on AoI in C-V2X.

### 4.1. Simulation Settings

According to the C-V2X standard, we use a 10 MHz bandwidth with a total of 50 RBs. The message size is set to 500 Bytes, and QPSK modulation is applied for propagation. The $T_{C}$ has a granularity of 100 ms between 100 ms and 1 s. The arrival rates $\lambda_{H}$, $\lambda_{D}$, and $\lambda_{M}$ for different message types, as shown in Equation (1), are set to 0.0001. The $T_{H}$ and $T_{D}$ are set to 100 ms and 500 ms, respectively. The $K_{H}$ and $K_{D}$ are set to 8 and 5, respectively. The transmission power of all vehicles is uniformly set to 23 dBm, and the speed is set to 120 km/h.

### 4.2. Performance Evaluation

The average AoI of packets in the queue can be expressed as a function of $\varphi_{i,n}^{t,b}$, as follows:

(13) $$\bar{\varphi }=\frac{1}{N_{v}}\frac{1}{N}\frac{1}{b}\sum_{i=1}^{N_{v}}\sum_{n\in N}\sum_{b}^{L}\varphi_{i,n}^{t,b}$$

where Nv represents the total number of vehicles in the scene, where N = 4 indicates four types of messages. The $\Delta^{t}$ is defined as the average of $\Phi_{i\to j}^{t}$ between each pair of vehicles.

(14) $$\Delta^{t}=\frac{1}{N_{v}}\frac{1}{N_{v}-1}\sum_{j=1}^{N_{v}}\sum_{i=1}^{N_{v}-1}\Phi_{i\to j}^{t}$$

where $\Phi_{i\to j}^{t}$ represents the AoI between vehicles *i* and *j* at time *t*, taking into consideration the impact of half-duplex communication. Thus, the summation involves calculating the mean AoI between different vehicle pairs *i* and *j*. In general, a higher $\Delta^{t}$ indicates a higher number of transmission failures.

Table 2 presents the communication success rate for different vehicle counts under number of vehicles and Γ, which represents the proportion of successfully received messages compared to all received messages. The vehicle density is set at 60 and 100 vehicles/km, and the length of load is set as 500 m. So it can be observed that the success rate is generally higher for 30 vehicles compared to 50 vehicles. This is because an increase in the number of vehicles enhances the probability of reserving resources at the same time, deteriorating resource contention. Furthermore, the success rate at Γ=20 ms is consistently lowest. This is because a smaller CSR will result in a higher probability of each resource being reserved by multiple vehicles. Moreover, it is worth noting that for a given number of vehicles, regardless of the value of Γ, employing NOMA technology consistently yields better transmission success rates compared to C-V2X with OMA.

**Figure 2:**
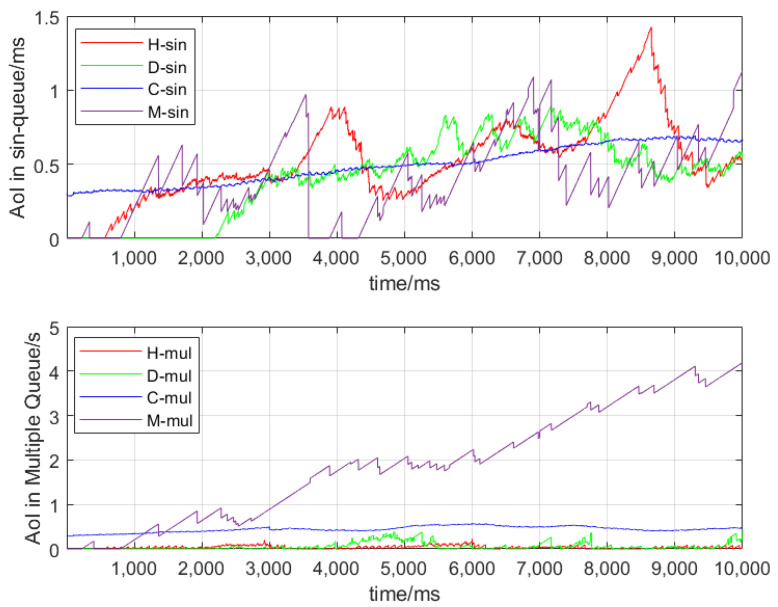
AvgAoI in different queues.

Figure 2 shows the AoI variation trend in various messages when the number of vehicles is set to 50 and Γ is set to 100 ms. The upper part of the graph shows the AoI change in information when a vehicle uses a single queue, while the lower part shows the situation when a vehicle uses a multi-priority queue. At this point, the messages in the queue will pile up, leading to an increasing AoI. It can be seen when there is only one queue, their overall trend in AoI change is similar. In a multi-priority queue, although the probability of generating MHD messages is the lowest, its AoI is the highest due to its lowest priority. So in multi-priority queues, the AoI of high-priority messages decreases to a certain extent, ensuring the timeliness of high-priority messages.

**Figure 3:**
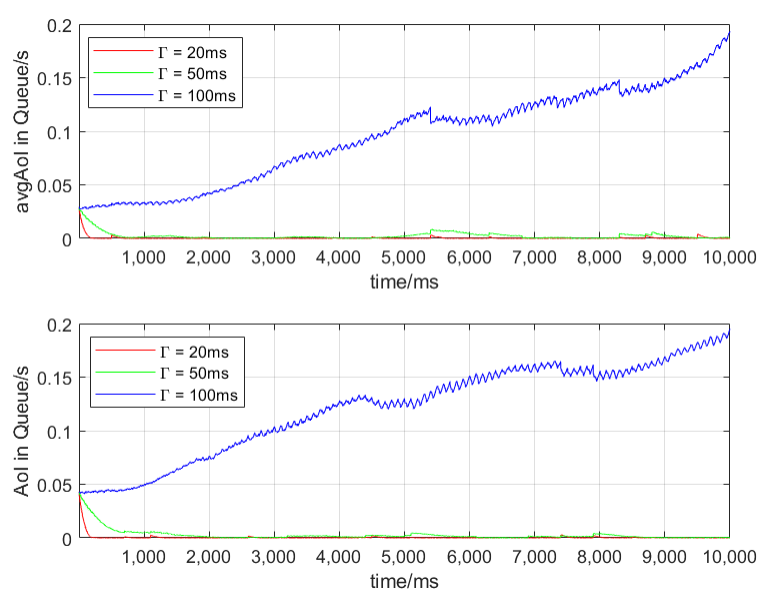
AvgAoI in queues.

Figure 3 presents the $\bar{\varphi }$ in multi-priority queues with different values of Γ when the number of vehicles is 30 and 50, respectively. When Γ is 20 ms, the inter-arrival interval of various types of packets is close to 100 ms (much larger than Γ), so the queue remains empty for most of the time; the $\varphi_{i,n}^{t,b}$ hardly increases. Similarly, when Γ is 50 ms, the $\bar{\varphi }$ is also relatively small. However, when Γ is 100 ms, which is greater than the packet inter-arrival interval, packets experience more time while waiting in the queue. In addition, it can be observed that when there are more vehicles, the value of $\bar{\varphi }$ increases to a certain extent compared to that of fewer vehicles.

**Figure 4:**
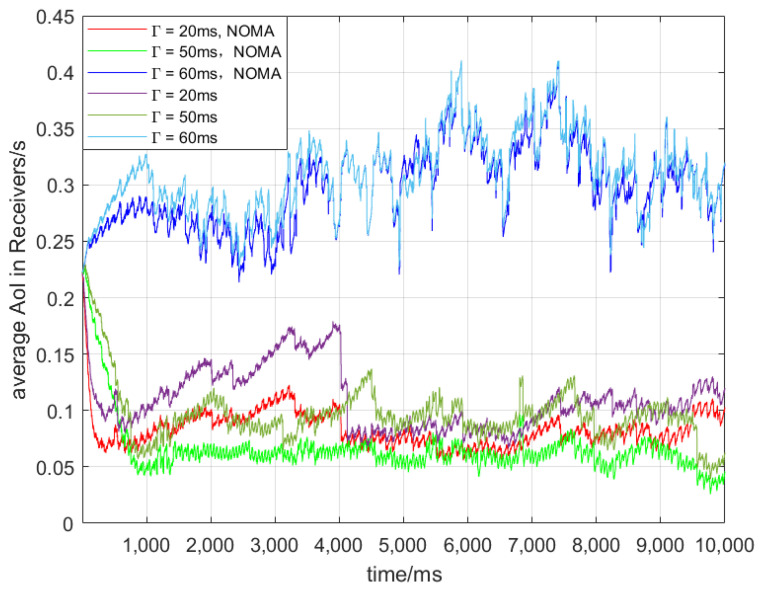
$N_{v}$ = 30.

Figure 4 shows the $\Delta^{t}$ of 30 vehicles with respect to different Γ. It can be observed that for different Γ, NOMA consistently decreases $\Delta^{t}$. As shown in Table 1, when Γ is 20 ms, the collision probability is relatively high, resulting in the shorter staying time of messages in the queue, but the AoI of the receiving end is not becoming smaller. By contrast, when Γ is 100 ms, the collision probability is lower. However, due to the accumulation of $\varphi_{i,n}^{t,b}$, it continues to rise and becomes the oldest AoI in the three situations. When Γ is 50 ms, the collision rate is the lowest among the three considered cases, and $\varphi_{i,n}^{t,b}$ does not accumulate, resulting in the lowest $\Delta^{t}$ throughout the entire observation period.

**Figure 5:**
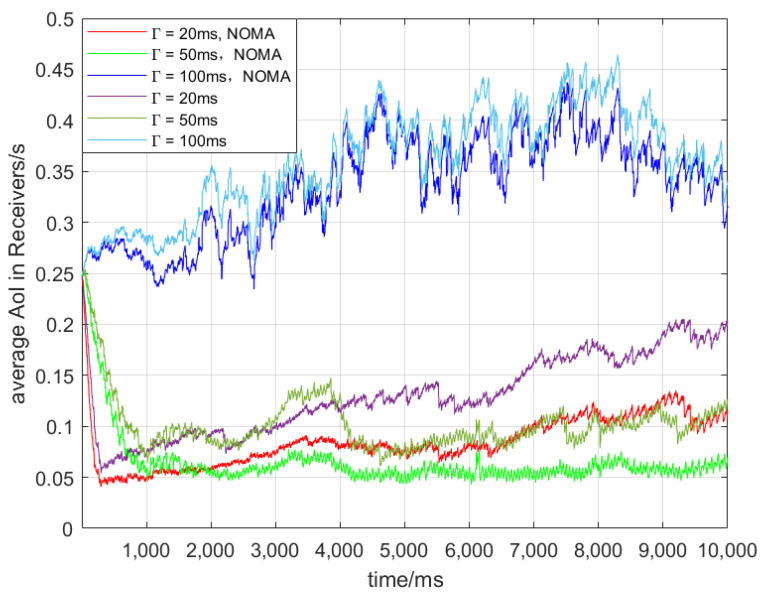
$N_{v}$ = 50.

Figure 5 depicts the $\Delta^{t}$ for 100 vehicles under different Γ. A comparison between Figure 4 and Figure 5 reveals that an increase in the number of vehicles leads to a general increasing trend for all cases, which is attributed to the rising collision probability with an increasing number of vehicles. Additionally, the trends in these two cases are similar to those shown in Figure 4, with the highest AoI at Γ = 100 ms and the lowest AoI at Γ = 50 ms. At 20 ms, communication suffers more interference, leading to higher Aol. At 100 ms, message accumulation in the queue causes delays and increased overload. The 50 ms interval selection can better balance these effects, resulting in a lower Aol. NOMA has positive impacts on AoI for different Γ.

## 5. Conclusions

This paper considers vehicle mobility and analyzes the impact of NOMA on the AoI in different scenarios. Firstly, we propose a novel multi-priority queue AoI calculation model for C-V2X communication. Then, we investigate the improvement in transmission success rate using NOMA based on SIC to observe changes in AoI. The following conclusions are drawn from our analysis:

- Under the same Γ, the collision probability varies with different vehicle counts. Adjusting Γ can reduce collision occurrences, but it must be carefully selected to avoid increased AoI.
- NOMA enhances SINR and reduces the impact of collisions, leading to decreased AoI in various scenarios in the C-V2X system.

## Figures and Tables

**Table 1:** The summary for notations.

Notation	Description	Notation	Description
RB	The time slot where vehicle reserves resources.	$T_{C}$	The generation interval of CAM messages.
$T_{D}$	DENM repeat transmission interval.	$T_{H}$	HPD repeat transmission interval.
$K_{D}$	Number of repeated transmissions of DENM.	$K_{H}$	Number of repeated transmissions of HPD.
$\lambda_{H,D,M}$	The new message arrival rates for the three message types: HPD, DENM, and MHD.	*L*	The maximum length of the message queue.
*q*	The queue length.	*s*	The transmission action.
SPS	The method for vehicles to autonomously choose communication resources.	SW	The value of the reselection counter for vehicles.
RC	The value of the reselection counter for vehicles.	RRI	Resource selection buffer time.
*b*	The position of the message in queue.	$\varphi_{i,n}^{t,b}$	AoI of messages at position *b* in queue *n* of vehicle *i*.
$\Phi_{i\to j}^{t}$	the AoI of the receiving end *j* for vehicle *i*.	$u_{i\to j}^{t}$	Indicates that *i* transmitted the message to *j*.
*Q*	The size of the transmitted message.	$R_{th}$	Transmission rate threshold.
$p_{i}^{t}$	The transmission power of vehicle *i*.	$\sigma^{2}$	Noise energy.
$P_{col}$	Resource collision probability.	π	The probability that the vehicle is at the moment when it is ready to select resources.

**Table 2:** Communication success rate in C-V2X.

$N_{v}$	30			50		
RRI	20	50	100	20	50	100
OMA	0.82891	0.83738	0.91560	0.75367	0.80050	0.85184
NOMA	0.89488	0.93332	0.97274	0.87902	0.92636	0.95356

## Data Availability

The data are contained within the article.

## References

[1] Fan J., Yin S., Wu Q., Gao F. Study on Refined Deployment of Wireless Mesh Sensor Network. Proceedings of the IEEE International Conference on Wireless Communications, Networking and Mobile Computing (WICOM’10).

[2] Zhang C., Xu X., Wu Q., Fan P., Fan Q., Zhu H., Wang J. (2024). Anti-Byzantine Attacks Enabled Vehicle Selection for Asynchronous Federated Learning in Vehicular Edge Computing. China Commun..

[3] Ji M., Wu Q., Cheng N., Chen W., Wang J., Letaief K.B. (2024). Graph Neural Networks and Deep Reinforcement Learning Based Resource Allocation for V2X Communications. IEEE Internet Things J..

[4] Qi K., Wu Q., Fan P., Cheng N., Chen W., Letaief K.B. (2024). Reconfigurable Intelligent Surface Aided Vehicular Edge Computing: Joint Phase-Shift Optimization and Multi-User Power Allocation. IEEE Internet Things J..

[5] Liu L., Chen C., Pei Q., Maharjan S., Zhang Y. (2021). Vehicular Edge Computing and Networking: A Survey. Mob. Networks Appl..

[6] Wu Q., Wang W., Fan P., Fan Q., Zhu H., Letaief K.B. (2024). Cooperative Edge Caching Based on Elastic Federated and Multi-Agent Deep Reinforcement Learning in Next-Generation Networks. IEEE Trans. Netw. Serv. Manag..

[7] Wu Q., Wang X., Fan Q., Fan P., Zhang C., Li Z. (2023). High Stable and Accurate Vehicle Selection Scheme based on Federated Edge Learning in Vehicular Networks. China Commun..

[8] Shao Z., Wu Q., Fan P., Cheng N., Fan Q., Wang J. (2024). Semantic-Aware Resource Allocation Based on Deep Reinforcement Learning for 5G-V2X HetNets. IEEE Commun. Lett..

[9] Qi K., Wu Q., Fan P., Cheng N., Chen W., Wang J., Letaief K.B. (2024). Deep-Reinforcement-Learning-Based AoI-Aware Resource Allocation for RIS-Aided IoV Networks. IEEE Trans. Veh. Technol..

[10] Zhang C., Zhang W., Wu Q., Fan P., Fan Q., Wang J., Letaief K.B. (2024). Distributed Deep Reinforcement Learning Based Gradient Quantization for Federated Learning Enabled Vehicle Edge Computing. IEEE Internet Things J..

[11] Gu X., Wu Q., Fan P., Fan Q., Cheng N., Chen W., Letaief K.B. (2024). DRL-Based Resource Allocation for Motion Blur Resistant Federated Self-Supervised Learning in IoV. IEEE Internet Things J..

[12] Wan S., Lu J., Fan P., Shao Y., Peng C., Letaief K.B. (2021). Convergence Analysis and System Design for Federated Learning Over Wireless Networks. IEEE J. Sel. Areas Commun..

[13] Dai Y., Xu D., Maharjan S., Qiao G., Zhang Y. (2019). Artificial Intelligence Empowered Edge Computing and Caching for Internet of Vehicles. IEEE Wireless Commun..

[14] Wang K., Yu F.R., Wang L., Li J., Zhao N., Guan Q., Li B., Wu Q. (2019). Interference Alignment with Adaptive Power Allocation in Full-Duplex-Enabled Small Cell Networks. IEEE Trans. Veh. Technol..

[15] Wu Q., Wang S., Ge H., Fan P., Fan Q., Letaief K.B. (2024). Delay-sensitive Task Offloading in Vehicular Fog Computing-Assisted Platoons. IEEE Trans. Netw. Serv. Manag..

[16] Fan P., Feng C., Wang Y., Ge N. Investigation of the time-offset-based QoS support with optical burst switching in WDM networks. Proceedings of the 2002 IEEE International Conference on Communications. Conference Proceedings, ICC 2002 (Cat. No.02CH37333).

[17] Molina-Masegosa R., Gozalvez J. (2017). LTE-V for sidelink 5G V2X vehicular communications: A new 5G technology for short-range vehicle to-everything communications. IEEE Veh. Technol. Mag..

[18] 3G Partnership Project (2017). Evolved Universal Terrestrial Radio Access (E-UTRA); Medium Access Control (MAC) Protocol Specification (v14.3.0, Release 14).

[19] Di B., Song L., Li Y., Li G.Y. (2017). Non-orthogonal multiple access for high-reliable and low-latency V2X communications in 5G systems. IEEE J. Sel. Areas Commun..

[20] Chen X., Lu J., Fan P., Letaief K.B. (2017). Massive MIMO Beamforming With Transmit Diversity for High Mobility Wireless Communications. IEEE Access.

[21] Zhang Y., Peng K., Chen Z., Song J. SIC vs. JD: Uplink NOMA techniques for M2M random access. Proceedings of the 2017 IEEE International Conference on Communications (ICC).

[22] Liu J., Xiong K., Ng D.W.K., Fan P., Zhong Z., Letaief K.B. (2020). Max-Min Energy Balance in Wireless-Powered Hierarchical Fog-Cloud Computing Networks. IEEE Trans. Wirel. Commun..

[23] Jiang R., Xiong K., Fan P., Zhang Y., Zhong Z. (2019). Power Minimization in SWIPT Networks With Coexisting Power-Splitting and Time-Switching Users Under Nonlinear EH Model. IEEE Internet Things J..

[24] Guo Y., Xiong K., Lu Y., Wang D., Fan P., Letaief K.B. (2021). Achievable Information Rate in Hybrid VLC-RF Networks With Lighting Energy Harvesting. IEEE Trans. Commun..

[25] Situ Z., Ho I.W.-H., Hou Y., Li P. The Feasibility of NOMA in C-V2X. Proceedings of the IEEE INFOCOM 2020—IEEE Conference on Computer Communications Workshops (INFOCOM WKSHPS).

[26] Hirai T., Murase T. (2020). Performance Evaluation of NOMA for Sidelink Cellular-V2X Mode 4 in Driver Assistance System With Crash Warning. IEEE Access.

[27] Dey U.K., Akl R., Chataut R. Performance Improvement in Cellular V2X (C-V2X) by Using Massive MIMO Jacobi Detector. Proceedings of the 2022 IEEE 19th International Conference on Smart Communities: Improving Quality of Life Using ICT, IoT and AI (HONET).

[28] Kaul S., Yates R., Gruteser M. (2012). Real-time status: How often should one update?. Proc. IEEE Infocom. Mar..

[29] Ge Y., Xiong K., Wang Q., Ni Q., Fan P., Letaief K.B. AoI-minimal Power Adjustment in RF-EH-powered Industrial IoT Networks: A Soft Actor-Critic-Based Method. IEEE Trans. Mob. Comput..

[30] Zheng H., Xiong K., Fan P., Zhong Z., Letaief K.B. (2021). Age of Information-Based Wireless Powered Communication Networks With Selfish Charging Nodes. IEEE J. Sel. Areas Commun..

[31] Peng F., Jiang Z., Zhang S., Xu S. (2021). Age of Information Optimized MAC in V2X Sidelink via Piggyback-Based Collaboration. IEEE Trans. Wirel. Commun..

[32] Mlika Z., Cherkaoui S. (2022). Deep Deterministic Policy Gradient to Minimize the Age of Information in Cellular V2X Communications. IEEE Trans. Intell. Transp. Syst..

[33] Akar N., Dogan O. (2021). Discrete-Time Queueing Model of Age of Information With Multiple Information Sources. IEEE Internet Things J..

[34] Zhang Z., Zhu X., Jiang Y., Cao J., Liu Y. Closed-Form AoI Analysis for Dual-Queue Short-Block Transmission with Block Error. Proceedings of the 2021 IEEE Wireless Communications and Networking Conference (WCNC).

[35] Liu X., Liu H., Zheng K., Liu J., Taleb T., Shiratori N. (2024). AoI-minimal Clustering, Transmission and Trajectory Co-design for UAV-assisted WPCNs. IEEE Trans. Veh. Technol..

[36] Wijesiri N.B.A. G.P., Haapola J., Samarasinghe T. A Markov Perspective on C-V2X Mode 4. Proceedings of the 2019 IEEE 90th Vehicular Technology Conference (VTC2019-Fall).

[37] Gonzalez-Martín M., Sepulcre M., Molina-Masegosa R., Gozalvez J. (2019). Analytical Models of the Performance of C-V2X Mode 4 Vehicular Communications. IEEE Trans. Veh. Technol..

[38] Haapola G.P.W.N.B.A.J., Samarasinghe T. (2021). A Discrete-Time Markov Chain Based Comparison of the MAC Layer Performance of C-V2X Mode 4 and IEEE 802.11p. IEEE Trans. Commun..

[39] Cecchini G., Bazzi A., Masini B.M., Zanella A. LTEV2Vsim: An LTE-V2V simulator for the investigation of resource allocation for cooperative awareness. Proceedings of the 2017 5th IEEE International Conference on Models and Technologies for Intelligent Transportation Systems (MT-ITS).

